# IgM Antibodies Can Access Cryptic Antigens Denied to IgG: Hypothesis on Novel Binding Mechanism

**DOI:** 10.3389/fimmu.2019.01820

**Published:** 2019-08-02

**Authors:** Eric Chun Yiu Law, Danny Tze Ming Leung, Frankie Chi Hang Tam, Kitty Kit Ting Cheung, Naomi Hua Yin Cheng, Pak Leong Lim

**Affiliations:** ^1^Clinical Immunology Unit, The Chinese University of Hong Kong, Hong Kong, China; ^2^IgGENE, FoTan, Hong Kong, China

**Keywords:** IgM, antibody specificity, guanosine triphosphate, lock-and-key, antigen-binding site, steric hindrance, skeletal muscle, auto-antibodies

## Abstract

Antibodies are well-known protein mediators of immunity. IgM is the primordial member and the neglected sibling of the later-evolved and more proficient IgG in regard to their therapeutic and diagnostic use. Serendipitously, however, we found a paradox: While murine IgM antibodies specific for guanosine triphosphate (GTP) were able to recognize native guanylyl antigens found in primate or rat muscle tissues by immunofluorescence assays (which mimicked the auto-antibodies from autoimmune patients to skeletal or smooth muscle), the murine and human IgG counterparts failed. The results were replicated in cell-free direct binding assays using small latex microspheres decorated densely with GTP. The IgG antibodies could bind, however, if GTP was presented more spaciously on larger particles or as a univalent hapten. Accordingly, oligomerization of GTP (30-mer) destroyed the binding of the IgG antibodies but enhanced that of the IgMs in inhibition ELISA. We reason that, contrary to current belief, IgM does not bind in a lock-and-key manner like IgG. We hypothesize that whereas the intact and rigid antigen-binding site of IgG hinders the antibody from docking with antigens that are obstructed, in IgM, the two component polypeptides of the antigen-binding site can dissociate from each other and navigate individually through obstacles like the ancestral single-polypeptide antibodies found in sharks and camelids, both components eventually re-grouping around the antigen. We further speculate that polyreactive IgMs, which enigmatically bind to more than one type of antigen, use the same modus operandi. These findings call for a re-look at the clinical potential of IgM antibodies particularly in specific areas of cancer therapy, tissue pathology and vaccine design, where IgG antibodies have failed due to target inaccessibility.

## Introduction

Nature designed the antibody molecule including a whole family of these immunoglobulins (Igs) with great intricacy and intrigue. Igs are indeed one of the most exhaustively studied bio-molecules and have over a century revealed an endless trove of invaluable molecular secrets. IgM is the primordial member and the first to respond to foreign insults to the body. It is produced rapidly to provide immediate and broad defense against infections. This broad coverage is facilitated by a low-affinity antigen-binding site. IgM antibodies can nevertheless agglutinate or opsonize multivalent antigens such as microorganisms because these Igs function as pentamers, equipped with a disposal of multiple antigen-binding sites to enhance the affinity (avidity). Each monomer has a pair of antigen-binding sites formed by the variable regions of the Ig heavy (VH) and light (VL) chain (1).

IgG is evolved later and the second isotype to appear in a systemic infection. It is derived from IgM through gene-rearrangement in the producer B cell which changes the constant region of the Ig heavy chain (CH). Concomitantly, the *VH* and *VL* genes, including those in the complementarity-determining region 3 of *VH* (HCDR3), undergo random somatic mutation which can structurally refine the antigen-binding site to allow better accommodation of the antigen (2, 3). With the increased affinity, IgG antibodies become more specific and efficient, and function as a monomer. The smaller size allows the protein to traverse into inter-vascular spaces denied to IgM. For these reasons, including the fact that it is more robust and easier to produce, IgG is preferred over IgM as the model molecule of study or as the designer antibody for therapy (4).

Our understanding of antibody specificity is indeed based largely on the IgG molecule. From the early studies of Landsteiner (5) to the later use of X-ray crystallography and protein modeling (6), the antigen-binding site has been conceived as a rigidified (rigid) structure which fits the antigen stereo-chemically like a lock-and-key (7). Most importantly, the antigen-binding site is pre-configured before antigen contact and stays in the same configuration always. Antigen is bound only when the antigen-binding site is able to dock with the antigen in the first place, and secondly, when there is sufficient physical complementariness between the two surfaces to allow short bonding forces to operate. The docking step itself has seldom been addressed (8) since the primary focus of the majority of binding studies is the end result of whether an antibody-antigen complex has been formed.

IgM antibodies are difficult to crystallize due to their large size (9) and not well studied, but are assumed to dock with antigen just like IgG antibodies i.e., via a rigidified, pre-configured antigen-binding site, in a lock-and-key manner. The antigen-binding site is believed to be more flexible than the IgG counterpart, but is nevertheless intact, formed by both VH and VL. This applies to both the antigen-induced immune IgMs and the natural IgM antibodies, the latter found normally at low levels in healthy people and animals (10), which are largely germline-encoded and produced by innate-like B lymphocytes (B1 and Marginal Zone B cells) (11, 12). The presumption also applies to the recently-discovered polyreactive (poly-specific) IgM antibodies which bind to more than one type of antigen (13). Many natural IgMs are in fact polyreactive, while most immune IgMs (derived from follicular B2 B cells) are mono-specific like the majority of IgGs. Polyreactivity is generally considered an oddity. Still an enigma, the general consensus is that the antigen-binding sites of polyreactive antibodies can constantly isomerize between one conformation and another before antigen contact (7, 14, 15), each isomer exhibiting a distinct antigen-binding site. Alternatively, after binding to the antigen, the antigen-binding site of these antibodies can adjust (“induce-fit”) to accommodate other antigens (7, 13, 15).

By serendipity, we re-visit the question of how IgM antibodies actually bind to antigen. We have worked with IgM antibodies for many years and were particularly impressed by the exquisite specificity of some of these e.g., a murine monoclonal antibody (mAb) to the O9 antigen found in *Salmonella* lipopolyssacharide (LPS) (16), and a murine mAb to guanosine triphosphate (GTP) (17). The anti-GTP mAb (Mab50) was also intriguing for another reason: It was suicidal for the producer cells under certain conditions (17). Earlier, we found an abundance of IgG auto-antibodies to GTP in a patient stricken with systemic lupus erythrematosus (SLE) (18) but could not assess the clinical significance of these. Potentially, GTP (or the guanylyl derivatives) is an important and vulnerable target in the body for auto-antibodies since it is found ubiquitously, notably, in the form of GTPases (19). These signaling proteins interact with the cell cytoskeleton comprising of the microfilaments (e.g., actin), intermediate filaments (e.g., cytokeratin), and microtubules (tubulin). Herein, we characterized Mab50 and other anti-GTP antibodies extensively in terms of their fine specificity and cellular targets. We stumbled on a stunning difference between the IgM and IgG antibodies: Only the former was able to bind to the guanylyl epitopes buried in muscle tissues. We confirmed this paradox using cell-free antigens in various experimental settings and consequently propose a radically new mechanism—different from lock-and-key—to account for the successful binding by the IgM antibodies. We further propose that polyreactive IgM antibodies are no different from immune IgM antibodies and these also use the same modus operandi.

## Materials and Methods

### Reagents and Antigens

Guanosine triphosphate (GTP), guanosine monophosphate (GMP), human serum albumin (HSA), bovine serum albumin (BSA), mouse serum albumin (MSA), and yeast transfer RNA (tRNA), were obtained from Sigma Chemical Co. (St. Louis, MO). Oligo G (30 mer) and oligo G (+C) (which contains 25 Gs and a 5′ CCCCC leader) were synthesized by Invitrogen (Carlsbad, CA). dGTP was also obtained from Invitrogen. Whole bacteriophage λ dsDNA (48,502 bp) was purchased from Amersham Biosciences (Buckinghamshire, UK), while ssDNA was prepared by boiling dsDNA in 100 μL buffer containing 10 mM Tris-Cl and 1 mM EDTA (pH 8.0) for 10 min and then quickly chilling it in ice water for >5 min.

Protein conjugates of GTP or GMP were made by covalently conjugating the hapten to HSA, BSA, or MSA according to Erlanger and Beiser (20). Briefly, the hapten (0.1 mM) was dissolved in 1 ml 0.1 M NaIO_4_ for 20 min at room temperature (RT). The albumin (5–10 mg), dissolved in 2 ml 0.1 M NaCO_3_/NaHCO_3_ buffer (pH 9.5), was added dropwise to the hapten and incubated for 1 h at RT with constant stirring. NaBH_4_ (5 ml, 20 mg/ml) was then added and incubated with stirring overnight at 4°C. The mixture was dialyzed against phosphate-buffered saline (PBS, 2 L) for 16 h at 4°C. The extent of hapten conjugation was estimated by comparing the absorbance at 253 nm of the conjugated protein (1 mg/ml) with that of the un-conjugated protein (1 mg/ml) in a UV spectrophotometer (Beckman DU 7500, USA). This difference in absorbance, when divided by the extinction coefficient for guanosine (Ec = 13,700 M^−1^ cm^−1^) and the molarity of the protein (assuming cuvette length of 1 cm), gives an estimate of the amount of hapten coated.

### Mouse Anti-GTP Antibodies

BALB/c mice were immunized with 50 μg GTP-HSA in complete Freund's adjuvant administered intraperitoneally on day 1 and boosted with 10 μg GTP-HSA in incomplete Freund's adjuvant intraperitoneally on day 14. Small samples of blood (0.2 ml) were obtained from the retro-orbital plexus and examined for antibody activity (ELISA) to GTP-MSA. Sera with high IgG anti-GTP titers were kept frozen for later use in the study. Responder mice were sacrificed for hybridoma production (17). Briefly, single spleen cells were prepared and chemically fused with NS0 myeloma cells (see below) using 50% (w/v) polyethylene glycol (mol wt 1,450 Da; Sigma) mixed with 10% DMSO. The fusion mixture was plated out at 1 × 10^6^ cells/ml in 96-well costar plates in RPMI 1640 medium (Invitrogen) supplemented with normal spleen (feeder) cells (1 × 10^7^ cells/ml). Clones that appeared were screened by ELISA against GTP-MSA. Antibody-secreting hybridomas were re-cloned by limiting dilution and expanded in culture, and in some cases, injected into BALB/c mice to produce ascites fluid (17). For some studies, Mab12 (IgG) was affinity-purified on protein-G Sepharose (Sigma) using 0.1 M glycine-NaOH (pH 11.0) as the eluting buffer; the protein content of the eluted material was determined using the BCA protein kit (Pierce, Rockford, IL), employing serially-diluted BSA as standards. In other studies, the purified Mab12 was biotin-conjugated using N-hydroxysuccinimido-biotin (Sigma–Aldrich, St. Louis, MO) freshly prepared in DMSO.

### Human Anti-GTP Antibodies

Several batches of sera from patients suspected of SLE sent to the Clinical Immunology Unit, Prince of Wales Hospital, Hong Kong, for the routine detection of anti-nuclear antibodies were recovered from the frozen (-80°C) bank and used in the present study. These were screened by ELISA for the presence of IgG and IgM antibodies to GTP. Sera with high levels of IgG antibodies (OD 450 nm > 2.5, 1:10 serum dilution) but low in IgM antibodies were selected and used. In some cases, the IgG antibodies were purified by affinity chromatography. Briefly, 1.0 ml serum was loaded on a 1 ml column containing GTP-BSA-conjugated Sepharose 4B beads (see below). The column was washed extensively with 30 ml of PBS buffer. The bound antibodies were eluted with 0.1 M glycine-NaOH buffer (pH 11) and the eluted material neutralized with 1.0 M Tris-HCL buffer (pH 2.4).

### VH and VL cDNA Sequencing

RNA was extracted from freshly-cultured cells using the RNeasy Mini Kit (QIAGEN Inc., Germantown, MD), from which cDNA was prepared using reverse transcriptase and random hexamers, both obtained from Invitrogen. *VH* and *VL* fragments were prepared from the cDNA using the following primers in polymerase chain reactions:

*Mab12 VH*, Forward primer: 5′-TGAGGTGCAGCTGGAGGAGTC-3′
*Mab12 VH*, Reverse primer: 5′-ACCAGGCATCCCAGGGTCACCATGGAGT- 3′
*Mab12 VL*, Forward primer: 5′-CAAATTGTTCTCACCCAGTCT-3′
*Mab12 VL*, Reverse primer: 5′-GGATACAGTTGGTGCAGCATC-3′
(Mab12 *VL* primers were obtained from Mouse IgG Library Primer Set 1 [Porgen Biotechnik, Heidelberg].)
*Mab23 VH and Mab50 VH*, Forward primer: Degenerate 5′ primer for leader sequences (Heavy Primer Mix [GE Healthcare Life Sciences, Chicago, IL])
*Mab23 VH and Mab50 VH*, Reverse primer: 5′-GAAGGACTGACTCTC-3′
*Mab23 VL and Mab50 VL*, Forward primer: Degenerate 5′ primer for leader sequences (Light Prime Mix [GE Healthcare Life Sciences, Chicago, IL])
*Mab23 VL and Mab50 VL*, Reverse primer: 5′-AAGACCTTAGAAGGGAAGATAGG-3′

The *VH* and *VL* fragments were sequenced using ABI PRISM BigDye^TM^ Terminators V3.1 (Perkin-Elmer, Foster City, CA) in an ABI PRISM 3730xl DNA Analyzer (Perkin-Elmer) (performed by Tech Dragon Limited, Shatin, Hong Kong). The IMGT/DNAPLOT program (The International Immunogenetics Database) was used to analyze the data.

### Cells and Cell Culture

The murine plasmacytoma cell line Sp2/0, the human epithelial cell line, HEp-2, and the human embryonic kidney cell line, HEK 293T, were all obtained from the American Type Culture Collection, Manassas, VA, USA, and maintained in RPMI 1640 medium containing 10% FCS, 100 IU/mL penicillin and 100 μg/mL streptomycin at 37°C and 5% CO_2_.

### Western Blot (WB) Analysis

Cell lysates were prepared from HEp-2 and HEK 293T cells by lysing the cells with buffer containing 1% NP-40, 150 mM NaCl, 10 mM Tris (pH 7.5), 1x protease inhibitor, and 1 mM phenylmethylsulfonyl fluoride (PMSF) (all from Sigma). After incubation for 30 min at 4°C, the lysate was centrifuged at 14,000 rpm (Eppendorf 5424, USA) for 10 min at 4°C. The supernatant obtained was reduced (95°C, 5 min) and resolved on 10–12.5% SDS-PAGE gel. The gel was transferred to PVDF membrane, which was then blocked with 2.5% BSA-PBS and later incubated with mAb12 (78 nmol/L [1:200 ascites fluid]), Mab23 (1:200 ascites), Mab50 (1:200 ascites) or anti-tubulin mAb (1:400 stock) at 4°C overnight. The membrane was incubated (2 h, RT) with HRP-conjugated goat anti-mouse Ig (whole molecule) (Sigma) and later developed using ECL chemoluminescence (Amersham Biosciences).

### Direct Enzyme-Linked Immunosorbent Assay (ELISA)

Immuno-II microtiter plates (Thermo Fisher Scientific, Massachusetts, USA) were coated with GTP-HSA, GMP-BSA or the carrier protein (100 μL, 25 ng/mL) in 0.1 M Na_2_C0_3_/NaHC0_3_ buffer (pH 9.6). After overnight incubation at 4°C, the wells were blocked with 2.5% BSA-PBS for 1 h at 37°C. The unknown antibody (100 μL, serially diluted in 2.5% BSA-PBS) was incubated in the wells for 30 min at RT, the wells washed with 0.02% Tween-PBS, and the assay developed using one of the following horseradish peroxidase conjugates: (a) Goat anti-mouse IgG+IgM+IgA-H&L (pre-adsorbed) (1:500, Abcam, Cambridge, UK), (b) Goat anti-mouse IgM (μ-specific) (1:5000, Abcam, Cambridge, UK), (c) Mouse anti-human IgG (1:2000, Thermo Fisher Scientific, Waltham, MA), (d) Goat anti-human IgM (μ) (1:2000, Thermo Fisher Scientific) or (e) Streptavidin (1:1000, Thermo Fisher Scientific). Following incubation (30 min, RT) and washing, the assay was developed using 3,3′,5,5′-tetramethylbenzidine (TMB) (100 μL, Clinical Science Products Inc., Massachusetts, USA) (10 min, RT) and the reaction stopped with 0.18 N H_2_S0_4._ The results were read at OD450 nm in a TECAN ELISA reader (Männedorf, Switzerland).

### Competition ELISA

Direct ELISA was first used to determine the half-maximal binding of the unknown antibody to the immobilized antigen (GTP-BSA; 550 ng/mL). Serial dilutions of the competing antigen (50 μL; see “Reagents and Antigens”) were then incubated (30 min, RT) with the antibody (50 μL, 2x concentration of half-maximal dilution). The wells were washed twice with 0.02% Tween-PBS, and following incubation with the developing antibody, the assay was developed as described in the direct ELISA. The results were plotted as titration curves.

Competition ELISA was also used to determine whether two antibodies share the same specificity. In this case, serial dilutions of the competing antibody (50 μL) were incubated (30 min, RT) with a pre-determined concentration of the primary antibody (50 μL) and the assay developed as described targeting the primary (indicator) antibody.

### Indirect Immunofluorescence Assay (IFA)

Single-cell smears of HEp-2 cells were obtained from Bio-Rad Laboratories (Berkeley, CA). Tissue sections of primate striated muscle or of rat stomach and kidney were obtained from Euroimmun (Lubeck, Germany) and used according to the manufacturer's instructions.

In each case, the cell smear or tissue section was first blocked with 2.5% BSA-PBS. The unknown antibody (30 μL, diluted in PBS) was incubated with the cell or tissue substrate for 30 min at RT. After washing, the preparation was incubated (30 min, RT) with one of the following fluorescein isothiocyanate (FITC)-conjugates used at 1:50 dilution: Goat anti-mouse Ig (whole molecule), goat anti-mouse IgG (γ-specific) or goat anti mouse IgM (μ-specific) (all obtained from Abcam), or goat anti-human Ig (whole molecule) (Zymed, San Francisco, CA). Following washing, the preparation was examined in a UV microscope (Eurostar, Euroimmun) and the images were captured using the ProgRes®CT3 microscope-camera and the ProgRes®CapturePro image-acquisition software (JENOPTIK Optical Systems GmbH, Jena, Germany). Reference antibodies to tubulin, cytokeratin, and tropomyosin were murine IgG mAbs obtained from Abcam, except for actin (Sigma). In some cases, the cell or tissue preparation was pretreated with proteinase K (2.5–5.0 μg in 1 mL 0.05M Tris-HCl [pH 8.0], Sigma) for 15 min at RT. In other cases, GMP-BSA (15 μL, 280 μg/ml) or free GTP (1 mM) was co-incubated with the unknown antibody. In the competition IFA used to determine whether two antibodies share the same HEp-2 specificity, the competing IgM mAb (Mab23 or Mab50; 15 μL) was incubated with the indicator IgG antibody (Mab12; 15 μL, 1:80 ascites) and the assay developed using FITC-conjugated goat anti-mouse Ig (γ-specific) (1:50 dilution).

Results were read visually by at least three people. In some cases, the fluorescence was quantified using Fiji Image J (https://imagej.net/Fiji). In smooth muscle, the same zone size was used to compare the fluorescence intensity between “m” and “p” in each tissue section. Similarly, the same zone size was used to compare the fluorescence between Mab50 and Mab12 in skeletal muscle.

### Absorption Studies Using Synthetic Antigen Constructs

GTP-adsorbents composed of magnetic latex particles (1 μm in diameter, carboxyl-modified; Merck Millipore, Ile-de-France, France) were conjugated with GTP-HSA (2.85 GTP residues per carrier) or GTP-MSA (9.16 GTP residues) using the active-ester coupling method (Thermo Scientific Technical Note TN-027.02; www.thermofisher.com.au). Briefly, the latex particles (1 ml, 1%) in coupling buffer (50 mM MES, 0.01% SDS, pH 6.1) was activated using N-(3-dimethylaminopropyl)-N′-ethylcarbodiimide hydrochloride (Sigma) (5 μl, 10 mg/ml, in H_2_O) and N-hydroxysulfosuccinimide (Sigma) (23 μl, 50 mg/ml, in H_2_O). The mixture was incubated on a roller-mixer (Multimix MM1, Luckham, UK) for 30 min at RT. The particles were centrifuged (1,500 rpm / 5 min [MIKRO 120, Hettich, Tuttlingen, Germany]), washed twice with coupling buffer and finally re-suspended in 1 ml coupling buffer. The GTP-albumin (1 ml, 0.16 μg/ml, in coupling buffer) was then added to the particle suspension and the mixture incubated on a roller-mixer for 1 h at RT. The particles were again centrifuged, washed twice with 1 ml 2.5% BSA-PBS and finally re-suspended in 0.5 ml 2.5% BSA-PBS.

GTP-adsorbents composed of Sepharose 4B beads (45–165 μm in diameter, CNBr-activated; GE Healthcare Life Sciences, MA, USA) were similarly conjugated with GTP-HSA or GTP-MSA (21). Thus, the beads (0.2 g dry weight) were swollen and activated with 1 mM HCL for 20 min at RT. The beads were centrifuged at 1,500 rpm for 5 min, washed twice with coupling buffer (0.1 M NaHCO_3_ /0.5 M NaCl, pH 8.3), re-suspended in 1.0 ml coupling buffer, and then incubated with the GTP-albumin (1.0 ml, 0.5 mg/ml in coupling buffer). The mixture was incubated on a roller-mixer at 4°C overnight and later centrifuged. The recovered beads were washed with excess coupling buffer and subsequently blocked with 0.1 M Tris-HCL (pH 8.0) for 1 h at RT. After washing, 10 mM HCL was added to the beads and incubated for 5 min at RT, the beads then washed extensively with PBS and finally re-suspended in 1 ml PBS.

Absorption was performed as follows: The unknown antibody was first titrated against GTP-BSA immobilized on a microtiter plate in a direct ELISA to find the half-maximal dilution. The antibody (120 μl, 2x concentration of half-maximal dilution) was incubated with the GTP-adsorbent (120 μl of 10% [w/v] Sepharose beads or 1% [w/v] latex particles) on a roller-mixer for 30 min at RT. The mixture was then centrifuged (1,500 rpm/5 min) and the supernatant (200 μl) recovered. The supernatant was then serially diluted and assayed for antibody against GTP-BSA in a direct ELISA. Control adsorbents included Sepharose beads or latex particles coated with glycine or BSA.

## Results

### Mouse mAbs and Affinity-Purified Human IgGs Recognize the Guanylyl Epitope in the GTP/GDP Molecule

**Figure 1:**
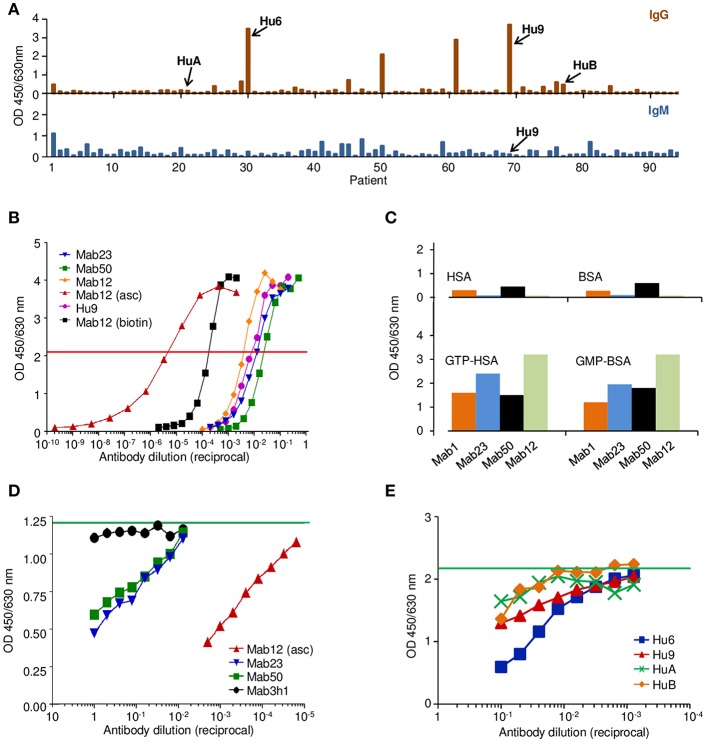
The anti-GTP antibodies derived from different sources differ in antibody concentration but recognize a common antigenic epitope. **(A)** IgG (top panel) and the corresponding IgM (bottom) ELISA to GTP-BSA of individual sera (1:100 dilution) from patients suspected of having SLE, activity expressed as absorbance units. Shown is one of several batches of sera used to select suitable specimens (e.g., Hu9) for the study. **(B)** ELISA titration of various antibody preparations used in the study, including the spent culture supernatants of Mab12, Mab23, and Mab50, as well as an ascites fluid preparation of Mab12 (asc), a preparation of biotin-labeled Mab12 (biotin), and an affinity-purified preparation of Hu9. Horizontal red line denotes the cut-off (50% maximal binding) used to estimate the antibody concentration based on a protein G-purified preparation of Mab12 (not shown). Mab12 (asc) has an antibody content of 16 μmol/L (2.5 mg/ml). **(C)** ELISA binding of various mAbs (1:10 dilution of culture supernatant, including Mab12) to GTP- or GMP-carrier conjugates or the carrier alone, expressed as absorbance units. **(D)** ELISA binding of biotin-labeled Mab12 (32 pmol/L) to GTP-BSA following co-incubation with serial dilutions of Mab23 (2.8 nmol/L, starting) or Mab50 (1.0 nmol/L). Other inhibitors used include Mab12 (asc) and the spent culture supernatant of an irrelevant hybridoma (3h1). Horizontal green line denotes the average reading of triplicate control samples (no inhibitor). **(E)** ELISA binding of biotin-labeled Mab12 (20 pmol/L) to GTP-BSA following co-incubation with serial dilutions of Hu9 and other human anti-GTP sera (see **A**; whole sera used). Horizontal green line denotes the average reading of triplicate control samples (no inhibitor).

We produced three IgM mAbs [Mab1, Mab23, and Mab50 (17)] and one IgG1 mAb (Mab12) from mice hyper-immunized with GTP-BSA. Clone Mab1 was inadvertently lost during the study and hence not used in some experiments. Serum from five of these mice (Mo1-Mo5) was also obtained for the study. In addition, we affinity-purified the anti-GTP IgG antibodies individually from nine patients (Hu1-Hu9) suspected of having SLE who had high levels of these antibodies (Figure 1A). Sera from two other patients (HuA and HuB) who had little or no IgG anti-GTP activity were also used without purification. The antibody concentration (8.1 nmol/L) determined of a purified preparation (Hu9) is similar to those of the typical spent culture supernatants of Mab12 (16.1 nmol/L), Mab23 (0.7 nmol/L) and Mab50 (0.3 nmol/L) (Figure 1B).

Preliminary investigations revealed that all the mAbs bound specifically to GTP or GMP (Figure 1C), although low-level binding to the carrier (BSA) was apparent with Mab1 and Mab50. The specificity of all antibodies examined was directed primarily at the guanine moiety since no major difference was found between GMP- and GTP-conjugates, nor between GTP and dGTP (see below), similar to the anti-guanosine antibodies described previously (22). In addition, by inhibition assay, Mab12 recognized an epitope shared by Mab23 and Mab50 (Figure 1D), as well as Hu9 (Figure 1E).

**Figure 2:**
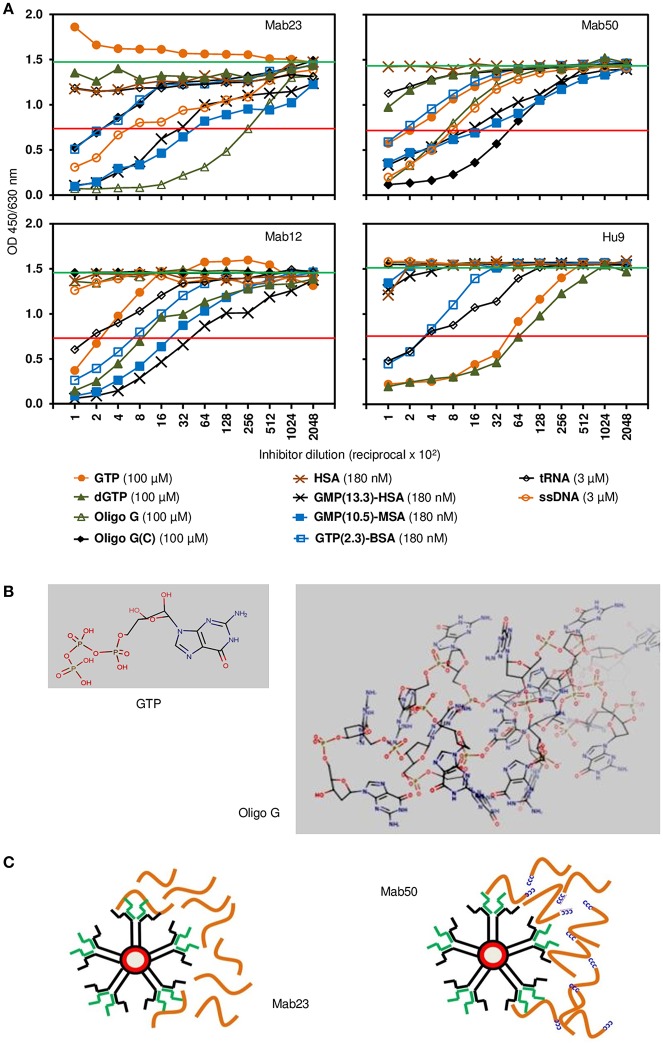
Competition ELISA reveals significant differences in fine specificity among the anti-GTP antibodies. **(A)** Resultant binding of the IgM antibodies (*top panel;* Mab23 [5.6 pmol/L], Mab50 [7.8 pmol/L]) or IgG antibodies (*bottom panel;* Mab12 [40 pmol/L], Hu9 [35 pmol/L]) to immobilized GTP-BSA following co-incubation with various soluble GTP or GMP analogs, each inhibitor serially diluted. The concentration of each inhibitor shown alongside the key symbols represents the first dilution. Also indicated is the no. of GTP/GMP residues per carrier molecule in the albumin conjugates. Horizontal green line (upper) denotes average reading of triplicate control samples (no inhibitor). Horizontal red line (lower) denotes 50% binding of antibody to the immobilized antigen. Data shown are all from one experiment and are representative of two-to-three experiments. **(B)** Chemical structures of GTP and oligo G (http://www.hmdb.ca/metabolites/HMDB01397) presented in 3D lowest-energy form (MarvinView [version 17.22.0], https://www.chemaxon.com/products/marvin) to illustrate the “open” or “cluttered” micro-environment, respectively, the guanylyl epitope can appear in. Only the proximal half of oligo G (30-mer) is shown. **(C)** Cartoon depicts an impression of the difference in binding between the two IgM mAbs: Mab23 prefers to bind to oligo G (orange-colored strings) whereas Mab50 prefers to bind to the modified oligomer that contains a CCCCC leader (blue-colored). Oligo G is just long enough for the two antigen-binding sites of an IgM monomer to bind to. In contrast, the CCCCC leader allows the molecules to form concatemers with one another generating antigenic topographies that presumably suits the pentameric binding of Mab50. Only the right half of each antibody is illustrated to show the interaction with antigen.

The fine specificity of two of the IgM mAbs (Mab23 and Mab50) and two IgG antibodies (Mab12 and Hu9) was examined more closely by competition ELISA using various GTP or GMP analogs as probe. All antibodies were pre-titrated and used at low antibody concentrations (5.6–40 pmol/L). The characterization results obtained are both interesting and instructive (Figure 2A). They reveal significant differences between the two isotypes, as well as, unexpectedly, within each isotype, based on the relative, overall affinity (avidity) each antibody has for a particular inhibitor. The relative avidity is defined in the present context as the concentration of inhibitor required to block 50% binding of the antibody with the immobilized antigen (23).

First, of the IgG antibodies, Mab12 bound modestly to the univalent GTP (400 nM) but better to dGTP (120 nM), while Hu9 bound extremely well to both of these haptens (16–21 nM). In contrast, with the IgMs, while Mab50 bound modestly to GTP (500 nM) and poorly to dGTP (>1 μM), Mab23 bound very poorly to both (>1 μM). Second, when GTP was polymerized in the form of oligo G, both the IgGs lost all binding, whereas in sharp contrast, Mab50 bound much better than before (140 nM), while Mab23 bound even better (18 nM). Furthermore, when a short cytosine leader was added to oligo G [oligo G(C)], this increased further the binding of Mab50 by 8-fold, but surprisingly decreased that of Mab23 (100-fold). The IgGs also failed completely to bind to this preparation.

Third, when the hapten (GTP or GMP) was conjugated in multiple copies to a protein carrier (BSA, HSA or MSA), both the IgMs bound well to these, the efficiency dependent on the hapten density. Thus, Mab23 bound ~ 10-fold better to both GMP-MSA (10.5 residues per carrier molecule; 35 pM) and GMP-HSA (13.3 residues; 56 pM) than to GTP-BSA (2.3 residues). Similar results were found for Mab50 but the avidity for GMP-MSA and GMP-HSA was 3-fold lower. Mab12 also bound as efficiently as Mab23 to these compounds, and poorly to GTP-BSA (300 pM). Hu9 also bound weakly to GTP-BSA (470 pM) but, surprisingly, not at all to GMP-HSA or GMP-MSA.

Fourth, similar to oligo G, ssDNA (single-stranded) was bound reasonably well by both the IgMs (4–5 nM) but not the IgGs (>30 nM). On the other hand, tRNA was bound weakly by the IgGs (8–20 nM) but not at all by the IgMs. With both isotypes (Mab12, Mab23 and Mab50), dsDNA (double-stranded), dATP, dCTP, dTTP, and (in a different experiment) thyroglobulin were not reactive (data not shown).

**Figure 3:**
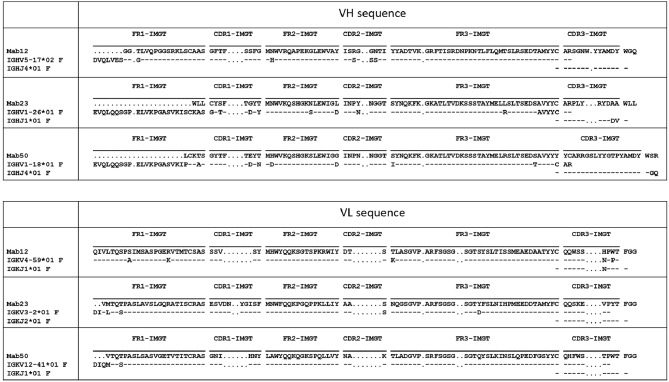
Both IgM mAbs are somatically mutated. Displayed are the amino-acid sequences of VH and VL of Mab23, Mab50, and Mab12, translated from the cDNA sequences and aligned with the nearest germline member according to IGMT/V-QUEST (www.imgt.org). Hyphen (–) indicates amino-acid identity, dot (.) indicates gap in sequence.

Different VH and VL genes were used by the three mAbs (see Figure 3 and Table 1). As expected, numerous non-silent mutations (5 mutations in VH, 0 in JH, 5 in VL) were found in Mab12. Surprisingly, both Mab23 (11, 2, 3 mutations, respectively) and Mab50 (11, 2, 2 mutations, respectively) were also extensively mutated. All three mAbs have long HCDR3s (18–23 amino-acids) while Mab12 and Mab50 share common DH and JH genes. SIFT analysis (Table 1) suggests that certain mutations in Mab50 VH (e.g., Ala105Tyr, Arg106Cys), Mab50 VL (Met4Val) and Mab23 VH (e.g., Lys24Trp, Gly27Cys) might be important in shaping (rigidifying) the antigen-binding site.

**Table 1:** Summary of amino-acid changes in VH and VL of Mab12, Mab23, and Mab50.

	**VH**							**DH**		**JH**		**HCDR3**
	**Family**	**FR1**	**CDR1**	**FR2**	**CDR2**	**FR3**	**CDR3**	**Family**		**Family**		**Length**
Mab12	IGHV5-17^*^02F	1 G11T	0	1 H40N	3 S58R S62G S63N	0	0	IGHD2-1^*^01	0	IGHJ4^*^01F	0	23
Mab23	IGHV1-26^*^01F	3 **K24W** **A25L** **S26L**	4 **G27C** T29S D36G Y38T	2 S49N D55L	1 N59Y	1 R92L	0	IGHD2	0	IGHJ1^*^01F	2 D116A V117A	18
Mab50	IGHV1-18^*^01F	2 P22L A25T	2 D36E N38T	2 D40H D55G	0	3 I66S T99S C104Y	2 **A105Y R106C**	F	0	IGHJ4^*^01F	2 G124S Q125R	21
	**VL**							**JL**				
	**Family**	**FR1**	**CDR1**	**FR2**	**CDR2**	**FR3**	**CDR3**	**Family**				
Mab12	IGKV4-59^*^01F	2 A9S K18R	0	0	0	1 K66T	2 N110H **P112W**	IGKJ1^*^01F	0			
Mab23	IGKV3-2^*^01F	2 L4M S7T	0	0	0	1 D86Y	0	IGKJ2^*^01F	0			
Mab50	IGKV12-41^*^01F	2 **M4V** S7T	0	0	0	0	0	IGKJ1^*^01F	0			

*Listed in the various gene segments of VH (including, DH and JH) and VL (including JL) are the non-silent mutations, if any, found in Mab12, Mab23, or Mab50, deduced from the nearest germline family member using IMGT/V-QUEST. Substitutions are designated by the germline residue (single alphabet) on the left, followed by the residue number, and then the mutated residue. Numericals denote the total number of mutations found for each segment, except those in HCDR3, which denote the total number of amino-acids present. HCDR3 comprises VH(CDR3), DH and JH. VH and VL substitutions considered as having a chance to affect protein function by SIFT (https://sift.bii.a-star.edu.sg/www/SIFT_seq_submit2.html) are marked in bold*.

### Both IgM and IgG Antibodies Stain the Cytoplasm of HEp-2 Cells

**Figure 4:**
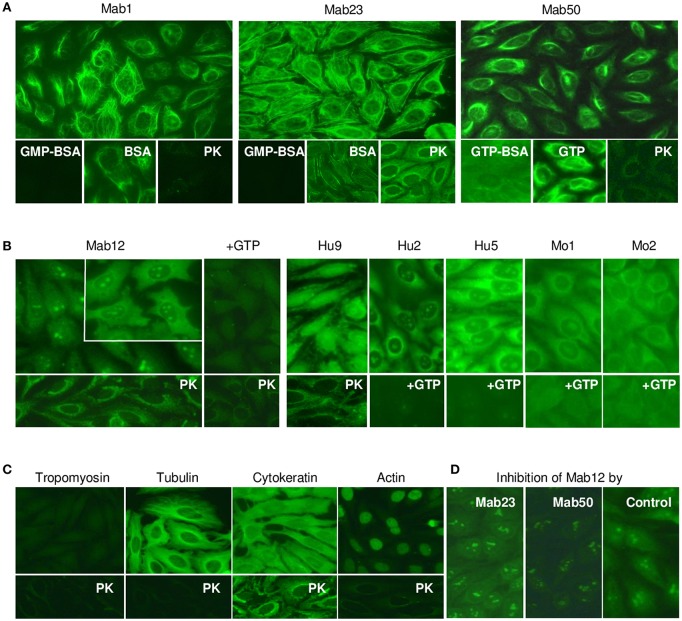
All the anti-GTP antibodies react with the cytoplasm of HEp-2 cells but subtle differences in staining are seen. **(A)** IFA reaction patterns of HEp-2 cells (x40 magnification) treated with the IgM mAbs (0.1–0.4 nmol/L), in some cases, in the presence of GMP-BSA, BSA, GTP-BSA, or GTP, or following cell-digestion by proteinase-K (PK). Data are representative of two-to-three separate experiments for each mAb. **(B)** Results of similar studies using the IgG antibodies: Mab12 (107 nmol/L), Hu9 (4.4 nmol/L), Hu1 and Hu2 (1:5 affinity-purified stock), Mo1 and Mo2 (1:10 whole serum). Inset in Mab12 used three-fold higher concentration of antibody. Data for Mab12 and Hu9 are representative of two-to-three experiments. **(C)** Results using murine reference antibodies (1:12.5 stock dilution) to various cytoskeletons, with or without PK treatment. **(D)** Effect of co-incubation with Mab23 or Mab50 (both~ 1 nmol/L) on the cytoplasmic staining by Mab12 (107 nmol/L); assay developed using IgG-specific antibody (This is a composite figure but the controls shown pertain to parallel experiments performed with the main experiment in each subset, with images captured under identical conditions).

Next, we compared the binding of the antibodies to native GTP antigens found in HEp-2 cells by IFA. All three IgM mAbs stained the cytoplasm with similar but distinctive patterns (Figure 4A). Specificity of binding was shown in all cases by the ability of GMP-BSA or GTP-BSA, but not GTP or BSA, to inhibit. PK-digestion of the cells destroyed the reactivity of Mab1 and Mab50 but allowed Mab23 to stain with a slightly different pattern (compared to untreated).

All fifteen IgG anti-GTP antibodies examined stained the cytoplasm similarly including the reactivity with the nucleolus (less distinct in Hu9); however, only six of these preparations are shown in Figure 4B. Unusually, Mab12 required exceedingly high concentrations of antibodies (>100 nmol/L or >25-fold higher than Hu9). In all cases examined, GTP inhibited both the cytoplasmic and nucleolar reactivity (13 cases, incompletely in some cases), while, following PK-treatment (3 cases), a new staining pattern similar to that of Mab23 (PK-treated) emerged. This pattern was also found when anti-cytokeratin antibodies were used (Figure 4C). This implicates cytokeratin as one of several antigens in HEp-2 recognized by these antibodies. Tubulin is another that is recognized by both IgM and IgG antibodies and which could be accessed without PK-treatment (Figure 4C). This explains why the cytoplasmic reactivity of Mab12, but not the nucleolar reactivity, could be blocked by Mab50 including, albeit marginally, Mab23 (Figure 4D).

**Figure 5:**
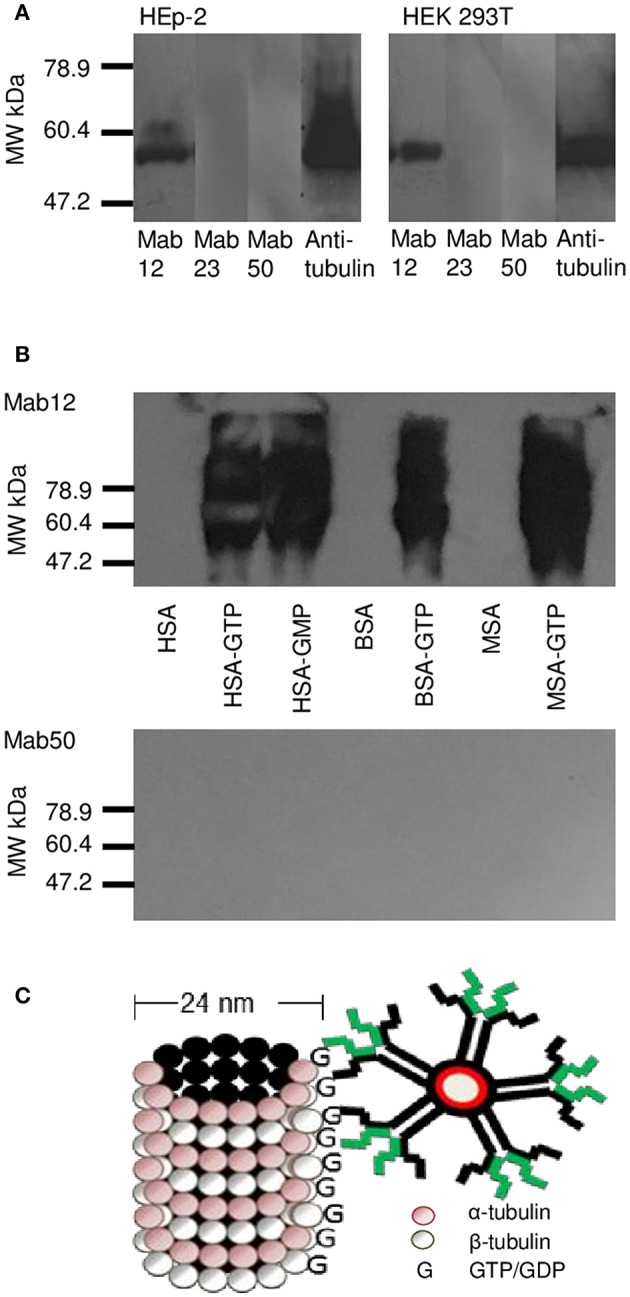
WB results reveal tubulin in cell lysates to be a target for Mab12. **(A)** Both Mab12 and anti-tubulin antibodies recognized a similar antigen in cell lysates derived from HEp-2 and HEK 293T. Mab23 and Mab50 are negative (Both composite blots obtained by image splicing and grouping). **(B)** Mab12 bound to various types of albumins conjugated with GTP but not to the carriers themselves. Mab50 did not bind to any of the carriers with or without GTP. **(C)** Cartoon illustrates partial structure of a microtubule, comprised entirely of α- and β-monomers of tubulin, each monomer (both 55 kDa in molecular weight) bearing a GTP/GDP molecule. The α-monomer-associated GTP/GDP, which is non-hydrolysable, is cryptic. Also shown in relative size is an IgM molecule.

WB analysis of the cell lysates obtained from HEp-2 and HEK 293T cells confirmed tubulin (the building block of microtubules) to be a target antigen for Mab12 (Figure 5A). The binding was specific as shown in parallel experiments using GTP- or GDP-conjugated albumins: Mab12 bound strongly to these but not the carrier albumins themselves (Figure 5B). (The blots showed varying degrees of self-aggregation of the albumins brought about by the conjugation). In contrast, neither Mab50 nor Mab23 bound at all to these cell lysates (Figure 5A) or, in the case of Mab50, the GTP-conjugates (Figure 5B).

### Only the IgM mAbs React With Skeletal and Smooth Muscle Tissues, Not the IgG Antibodies

**Figure 6:**
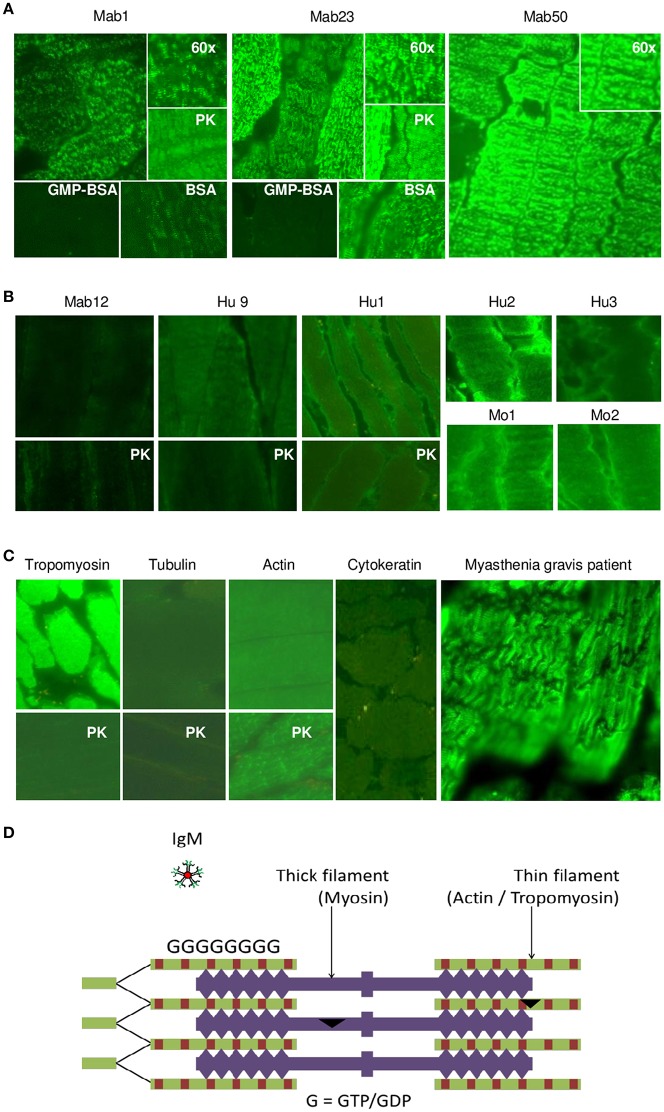
All three IgM anti-GTP mAbs but none of the IgG antibodies bind to skeletal muscle tissue. **(A)** IFA results (x40 magnification) of skeletal muscle tissue treated with the IgM mAbs (0.1–0.4 nmol/L), in some cases, in the presence of GMP-BSA or BSA, or following cell-digestion by PK. Inset shows higher magnification (x60). Data for Mab23 and Mab50 are each representative of at least three separate experiments. **(B)** Results of similar studies (including similar assay and viewing conditions) using the IgG antibodies: Mab12 (202–807 nmol/L), Hu9 (4.4 nmol/L), Hu1, Hu2 and Hu3 (1:5 affinity-purified stock), and Mo1 and Mo2 (1:10 whole serum; note the high background fluorescence). Data for Mab12 and Hu9 are representative of two-to-three separate experiments. **(C)** Results using murine reference antibodies to various cytoskeletons (with or without PK- digestion), including a serum from a *Myastenia gravis* patient. **(D)** Cartoon of cross-section of skeletal muscle showing the abundance of GTP/GDP molecules (G) in the actin-tropomyosin complex (thin filament), which forms a multi-layer network, and an IgM molecule in relative size (This is a composite figure but the controls shown pertain to parallel experiments performed with the main experiment in each subset, with images captured under identical conditions).

We next examined tissue sections of skeletal and smooth muscle by IFA and found a dramatic difference between the isotypes. Impressively, all three IgMs reacted strongly with skeletal muscle producing a striation pattern characteristic of the staining by true anti-skeletal muscle antibodies (Figure 6A). Binding was specific as shown by inhibition with GMP-BSA (BSA as control) in both of the cases examined (Mab1 and Mab23). In addition, PK-digestion had no effect on the binding.

In stark contrast, none of the fifteen IgG antibodies used—including high concentrations (200–800 nmol/L) of Mab12—were reactive; six of these are shown in Figure 6B. None produced the muscle striation and, based on quantitative fluorescence, Mab12 was 7-fold lower in intensity than Mab50. In all three cases examined (Mab12, Hu9 and Hu1), the antigen(s) could not be retrieved using PK-digestion. Reference IgG mAbs to tropomyosin and actin (especially after PK digestion) reacted strongly (Figure 6C), while antibodies to tubulin or cytokeratin were un-reactive. By comparison, the anti-skeletal muscle IgG antibodies from a *Myasthenia gravis* patient produced the characteristic striation pattern (Figure 6C).

**Figure 7:**
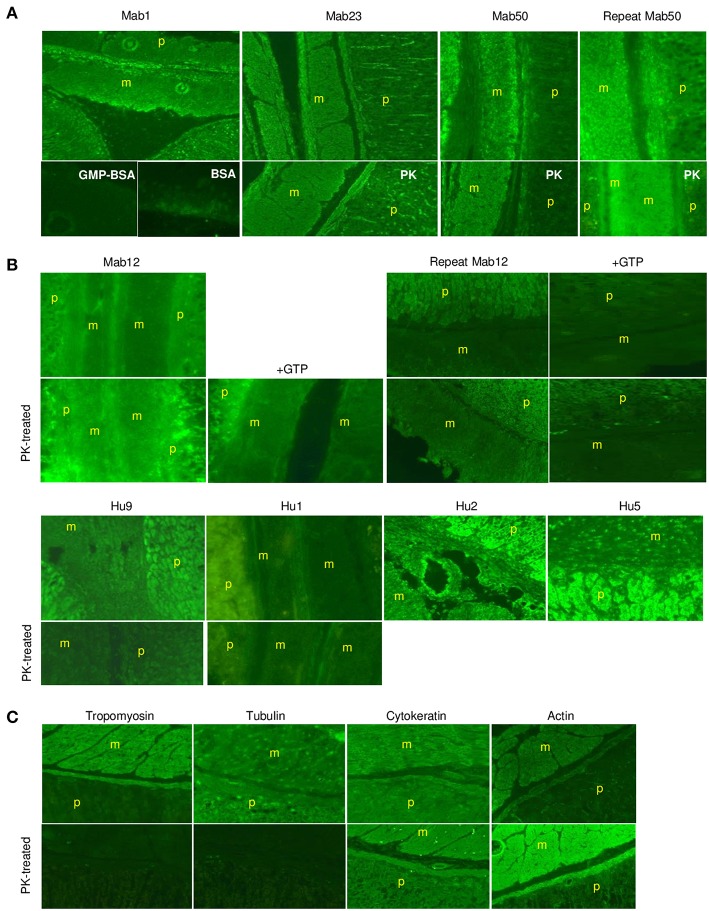
The IgM anti-GTP mAbs but not the IgG antibodies bind to smooth muscle tissue. **(A)** IFA results (x20) of smooth muscle tissue treated with the IgM mAbs. Conditions used are as for skeletal muscle. In some cases, the tissue was PK-treated or co-incubated with GMP-BSA or BSA. The *muscularis externa* (m) and parietal cells (p) are marked accordingly. Data for Mab23 and Mab50 are each representative of at least three experiments; replicate results shown for Mab50 were performed on separate occasions by different individuals. **(B)** IFA results (x20) of similar studies using the IgG antibodies. Conditions are as described for skeletal muscle, including the inclusion of GTP as inhibitor (GTP) or the use of PK-digestion in some cases. Two independent experiments performed separately by different individuals are presented for Mab12; in each, the various examinations were undertaken in parallel using the same diluted reagents and the results processed in exactly the same manner. Results for Hu9 and Hu1 (with and without PK treatment performed simultaneously) and other human purified IgGs (Hu2 and Hu5) represent one-to-three experiments. **(C)** IFA results of similar studies using murine reference antibodies to the various cytoskeletal networks (This is a composite figure but the controls shown pertain to parallel experiments performed with the main experiment in each subset, with images captured under identical conditions).

Similarly, in smooth muscle, all three IgM mAbs strongly stained the *muscularis externa* (marked “m” in illustration) in the tissue section even after PK digestion (Figure 7A), but none of the five IgG antibodies, including high concentrations of Mab12, were reactive (Figure 7B). Both isotypes, however, stained the adjacent parietal cells (marked “p”). Only the reactivity with the *muscularis externa* is relevant, and this is best judged against that of the parietal cells in each tissue section to avoid differences in imaging among sections. These results, in fact, can be objectively scored as a m/p ratio based on the relative fluorescence found between the two sites in each tissue section. Thus, while all the IgMs have m/p values above 1.0 (1.23 [Mab1], 1.20 [Mab23] and 1.42–1.61 [Mab50]), all the IgGs have values lower than 1.0 (0.61–0.78 [Mab12], 0.86 [Hu9], 0.34 [Hu1], 0.75 [Hu2] and 0.62 [Hu5]). Interestingly, following PK pretreatment (lower panel, whole row, Figure 7B), Mab12 (807 nmol/L), but not Hu9 or Hu1, could now bind to the *muscularis externa*. Although this reactivity was weak (m/p = 0.90, compared to 0.78 without PK-digestion), the observation was reproduced (m/p = 0.68, compared to 0.61) by different researchers (DTML and FCHT) and the reaction was specific (GTP-inhibitable). The main antigenic structure targeted is probably actin (or, less likely, cytokeratin) based on the reference antibodies used, since this is PK-resistant (lower panel, Figure 7C) and has a staining pattern (Figure 7C) resembling that of the IgMs.

### Similarly, Only the IgM mAbs React Well With High-Density Antigen Constructs

**Figure 8:**
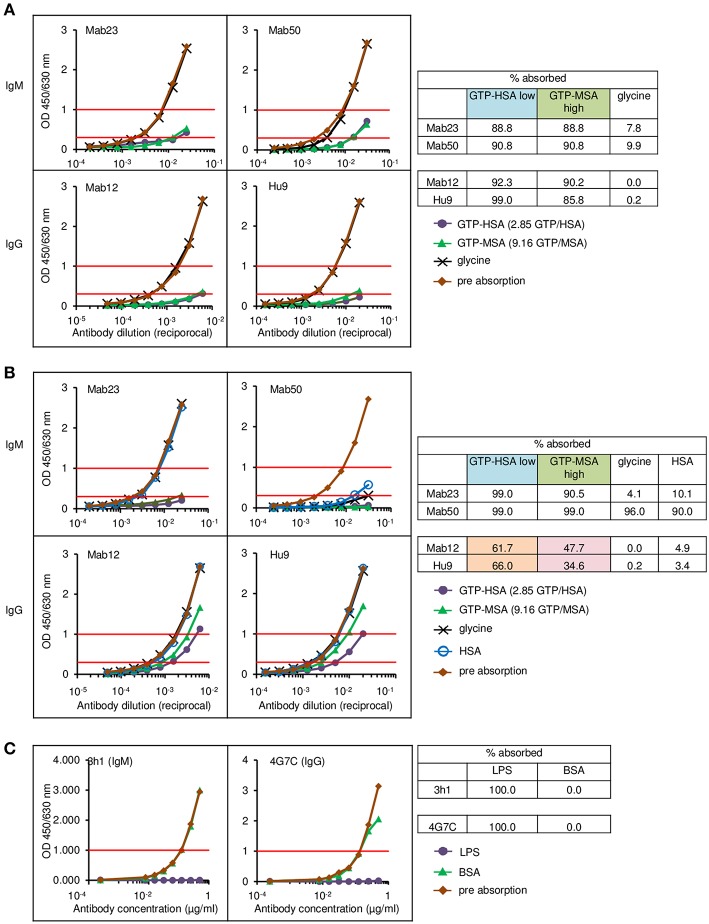
Direct cell-free demonstration showing how antigen “cluttering” can affect the binding of the IgG anti-GTP antibodies but not the IgM mAbs. Each antibody was incubated with each of the four antigen adsorbents and the binding efficiency determined by titrating the unbound antibody. Data represent two-to-three experiments. **(A)** ELISA results for the Sepharose bead experiments. The residual antibody activity is determined at OD 1.0 nm (horizontal red line, upper) wherever possible, or at OD 0.3 nm (horizontal red line, lower); numericals in key denote no. GTP residues per carrier protein. Table alongside summarizes the antibody activity absorbed (%) under different conditions; “low” or “high” denotes low or high GTP density, respectively. **(B)** ELISA results for the latex particles. Interpretation as in **(A)**. The brown and pink boxes highlight the inhibitory effect of antigen “cluttering” on the two IgGs. **(C)** ELISA results for the latex particles coated with Salmonella O9 LPS, showing equal absorption of an IgM mAb and an IgG mAb, both specific for O9.

To rule out tissue artifact, we next created artificial antigen constructs with different hapten densities. Thus, small latex microspheres (1 μm diameter) or large Sepharose beads (45–165 μm) were covalently coupled with GTP-HSA (2.85 GTP residues per carrier) or GTP-MSA (9.16 residues). The different preparations were used as adsorbents to compare the binding efficiency of two IgMs (Mab23 and Mab50) and two IgGs (Mab12 and Hu9), all used at low concentrations, including Mab12 (40 pmol/L). Results were scored based on the amount of antibodies left unbound following the absorption. Expectedly, the two isotypes bound differently. Both the IgMs bound well to all preparations, marginally better to the latex particles (91–99%) (Figure 8B) than to Sepharose (89–91%) (Figure 8A). Binding was highly specific in the case of Mab23 but not Mab50, the latter also binding extensively to control glycine-coated [96%] or HSA-coated [90%] latex particles. In contrast, while both the IgGs bound extremely well to the Sepharose adsorbents (90–99%) (Figure 8A), they bound only modestly to the latex particles coated with GTP-HSA (62–66%), and very poorly to particles coated at the higher GTP density (35–48%) (Figure 8B). Binding was highly specific with both IgGs shown by the fact that control glycine- or HSA-coated adsorbents were not bound at all.

For comparison, we performed similar binding studies using a different antigen, *Salmonella* LPS O9, also coupled to latex particles. Here, at the concentration of antigen used, the O9-specific IgG mAb bound as efficiently (100%) as the IgM mAb (Figure 8C).

## Discussion

The main thrust of our findings: IgM antibodies can bind to cryptic antigens, IgG cannot. This revelation is unexpected and important. It raises hope for translational medicine, and raises fundamental questions in biology. It also challenges the current concept on how IgM antibodies bind to antigen. While lacking formal proof, we herein posit that IgM antibodies use an unorthodox and presumably vestigial mechanism to dock with antigens. This is not about whole molecules navigating through crevices but rather, the ability of the individual antigen-binding sites to wriggle through obstacles to dock with the antigenic epitope recessed in the immediate vicinity.

We also found the converse, which is already well-known: IgG can bind to a single antigenic epitope, IgM cannot. Due to their low intrinsic affinity for GTP, both Mab23 and Mab50 require multivalent anchorage. Accordingly, these antibodies bound poorly to the univalent hapten in solution and particularly in cell smears or tissue sections where the hapten has to compete against high densities of cellular GTP/GDPs. These epitopes are found in the microtubules of HEp-2 cells, for example, which, in the native state, are readily bound by the IgMs (see Figure 5C). When these microtubules are denatured as individual tubulin monomers, however, the IgMs failed to bind. This, we contend, is because the linearized protein has only a single copy of GTP derived from the α-monomer (Figure 5C) (24). Similarly, when the various GTP/GMP-associated albumins are linearized in WB, IgM is not reactive because the GTP or GMP molecules (at most, 13 residues per carrier) become too spread out from one another, unlike the situation in the intact conjugate where multiple epitopes are brought close together. A similar explanation accounts for the failure of the IgMs to bind to tRNA, which consists mostly of double-stranded RNA except for an occasional but well-exposed GMP residue in the single-stranded hairpin loops.

In accordance, when GTP was polymerized as a 30-mer, both the IgMs could now bind. Both could similarly bind to ssDNA which has multiple GMPs distributed randomly along its length. More instructively, when the oligomer was modified so that it could circularize or form concatemers, the binding of Mab50 improved dramatically. The opposite, however, was found for Mab23. An analogous situation is, Mab50 inhibited Mab12 in HEp-2 cells much more efficiently than Mab23, whereas both mAbs were equally effective in the ELISA. This suggests that Mab23 recognizes a different antigenic topography from Mab50. A possibility is that whereas Mab50 binds like an inverted mushroom (25) involving the full circle of antigen-binding sites, Mab23 appears to employ just the pair from a single monomer (Figure 2C), similar to the bivalent (homotypic) binding that some IgG antibodies use to neutralize viruses (26, 27). This idiosyncratic binding may explain why Mab23 can bind to cytokeratin in HEp-2 cells, like the IgGs. Whether the difference is due to Mab23 having a 3-fold higher avidity or that Mab50 is marginally polyreactive (it can presumably crossreact with the polysytrene coat of latex particles) is unclear. Altogether, it seems possible that there are distinct sub-types of IgM.

The study involving native antigens in muscle tissue was most revealing. All three IgMs bound specifically to skeletal muscle and all three stained with the same characteristic pattern that resembled the binding by true anti-skeletal muscle auto-antibodies (28). This molecular mimicry—unknown previously—can lead to the false diagnosis of *Myastenia gravis*. These two sets of antibodies bind to different targets. The anti-GTP antibodies react with RAD GTPases in the thin filament while the anti-skeletal muscle antibodies react with myosin in the thick filament (Figure 6D). In both types of filament, the individual components exist as layers so that both types will result in a similar striation pattern when stained. The evidence showing RAD as the target is indirect: The thin filament is embroidered abundantly with this GTPase (29). Thus, RAD is found at the C-terminal end of short (40 nm) tropomyosin building blocks arranged head-to-tail in a double helix around a chain of actin monomers. There is thus an abundance of RAD in the thin filament within the wingspan (30–66 nm) (30) of an IgM for the antibody to bind to multivalently. The actual antigen recognized is the GTP/GDP molecule inside RAD. Since the tissue sections used have been formalin-fixed, it is likely that the hapten becomes immobilized and exposed (Even then, it seems it is still inaccessible to the IgGs; see later). In smooth muscle, the target antigen is presumably Rho, which is associated with myosin (31), cytokeratin (32), or actin (33).

IgG was evolved probably to complement IgM. Thus, both Mab12 and Hu9 have no problem in accommodating univalent antigens such as linearized tubulin, tRNA and, presumably in the HEp-2 nucleolus, U3 snoRNA (which has a similar structure to tRNA). However, there is a trade-off for the affinity (and specificity) achieved, which is of greater significance in our study: The binding becomes too restrictive. A particularly telling example is the inability of both Mab12 and Hu9 to bind to oligo G—the opposite of Mab23 and Mab50 (Figure 2B). This suggests the likelihood that the antigen-binding sites of these somatically-mutated antibodies are locked in a fixed configuration, and it is this inflexibility that prevents the antibody from docking with an epitope that is sterically blocked by neighboring structures. This explains why ssDNA, too, was not bound. This also explains why Hu9 could bind to GTP-BSA, which has only 2.3 GTP residues per carrier, but not GMP-HSA nor GMP-MSA, both with >10 GMP residues. Paradoxically, however, Mab12 bound better to the higher density conjugates. This presumably reflects the 20-fold lower affinity of the antibody for GTP compared to Hu9, and underscores the enormous micro-heterogeneity among antibodies even to a small hapten. It also seems the GMPs in both the conjugates are more spaced out than those in oligo G.

The IgG binding problem was particularly pronounced with native tissue antigens. Unlike the IgMs, none of the IgG antibodies examined reacted with skeletal or smooth muscle tissues. A possible obstacle hindering these antibodies in skeletal muscle is the phosphate-binding loop in RAD (34). In smooth muscle, the target antigen could be partially unmasked by PK-digestion which allowed Mab12 to bind to subsequently. Pertinently, Mab12 and the IgMs recognized a common epitope. Since the adjacent parietal cells in the tissue section were stained, this rules out any blocking artifact that might arise from tissue-fixation. All the polyvalent IgGs stained HEp-2 cells readily, probably because the target antigens are abundantly and openly displayed in the alcohol-fixed single cells; Mab12 is unusual in requiring high concentrations of antibodies.

The most unambiguous demonstration of the binding handicap comes from the cell-free study using small latex particles coated with GTP-albumin conjugates. Both Mab12 and Hu9 bound poorly to these preparations particularly at the higher hapten density. Both antibodies bound well, however, to the same conjugates, regardless of hapten density, coated on the larger Sepharose beads. Since the maximal coating density of the conjugates in the latex particles is 188 times higher than that of the Sepharose beads (Table 2), we argue the binding was hampered by the “cluttering” of antigens in the latex particles. This “cluttered” environment, however, did not pose any problem to Mab23 (results for Mab50 are invalidated by non-specific binding). It is noteworthy that not all antigens presented on the small latex particles are necessarily “cluttered” e.g., the *Salmonella* O9 antigen which exists abundantly as the terminal sugar of long trisaccharide repeats in LPS (35) is readily accessed by both IgG and IgM antibodies.

**Table 2:** Calculation of the maximum coating density of BSA on Sepharose beads and magnetic-latex particles.

	**Sepharose beads**	**Latex particles**
Mean diameter of particle	90 μm	1.1 μm
Surface area of particle	25.4 × 10^3^ μm^2^	3.8 μm^2^
No. particles per ml	2.6 × 10^9^	1.4 × 10^11^
Coupling capactity of BSA per ml of particles	10 mg	14.8 mg
No. BSA molecules that can be coupled per ml of particles	9.3 × 10^16^	1.4 × 10^17^
No. BSA molecules that can be coupled per particle	9.3 × 10^16^ / 2.6 × 10^9^ = 35.6 × 10^6^	1.4 × 10^17^/1.4 × 10^11^ = 1.0 × 10^6^
No. BSA molecules that can be coupled per surface area (μm^2^)	35.6 × 10^6^/25.4 × 10^3^ = 1.4 × 10^3^	1.0 × 10^6^/3.8 = 263 × 10^3^

*Based on information relating to coupling capacity and particle size supplied by the manufacturers (Merck Millipore, Ile-de-France, France, and GE Healthcare Life Sciences, MA, USA)*.

There are other accounts of target inaccessibility by IgG antibodies from other studies. First, there is the case of phosphorylcholine (PC) (36), a hapten found naturally in several microorganisms, including *Trichinella spiralis*. We found an IgM (MabTS2) and an IgG (Mab2) mAb from *T.spiralis-*infected mice, both highly specific for PC. However, whereas MabTS2 bound well to all types of PC-associated antigens, Mab2 could bind only to the immunizing *T.spiralis* antigen. We reconstructed Mab2 as a single-chain Fv (VH-VL) protein, but this destroyed the carrier-specificity. In contrast, Fab fragments made of the antibody, which included the first domain of CH and CL in addition to Fv, remained carrier-specific (37). We argued, therefore, that CH1 was crucial to the carrier-specificity of the antibody. An analogous situation is the important role the neighboring CDRs reportedly play in stabilizing the HCDR3 structure in the PGT145 antibody (38).

Second, there are numerous reports of failed IgG antibodies in tissue pathology. Invariably, the diagnostic probe employed is an IgG antibody rather than IgM because this generally has a higher affinity and specificity, and a perceived greater tissue penetrability. Often, too, the antibody binds well to the purified cell-free antigen but fails when it comes to the tissue section. The problem is often attributed to antigen masking by cellular structures or the effect of chemical fixation, which can sometimes be solved by treating the tissue with enzymes or heat (39). A whole variety of targets are affected but nuclear antigens are particularly notorious (40, 41). We suspect IgM antibodies may be a solution for some of these situations.

Third, IgG antibodies are also known to have failed in vaccine design and cancer immunotherapy. For example, IgG antibodies specific for the gp120 and gp41 envelop-spike antigens in human immunodeficiency virus (HIV-1) are known to be protective but the problem is that certain strategic sites of these antigens (e.g., V1V2 and MPER) can become blocked by glycans of host origin, rendering these antibodies ineffective (42). However, a small fraction of the antibodies, coined “broadly neutralizing antibodies,” manage to penetrate the glycan shield and reach the target through unusually long HCDR3 projections (38, 43). We anticipate that naturally-produced IgM antibodies can be found which can overcome such barriers and seek out a multitude of gp120 or gp40 epitopes despite the sparseness of these (26).

Fourth, obstruction can also come in a different form in situations of high antigenic density. The bound IgG molecule can itself hinder other IgG molecules (but not IgM) from binding to the neighboring sites. This explains why IgG antibodies can sometimes fail to neutralize viruses effectively due to the inadequate occupancy of target sites (44), or, similarly as we now understand, why they fail to compete against IgM antibodies in certain antibody-detection assays such as TUBEX (45).

**Figure 9:**
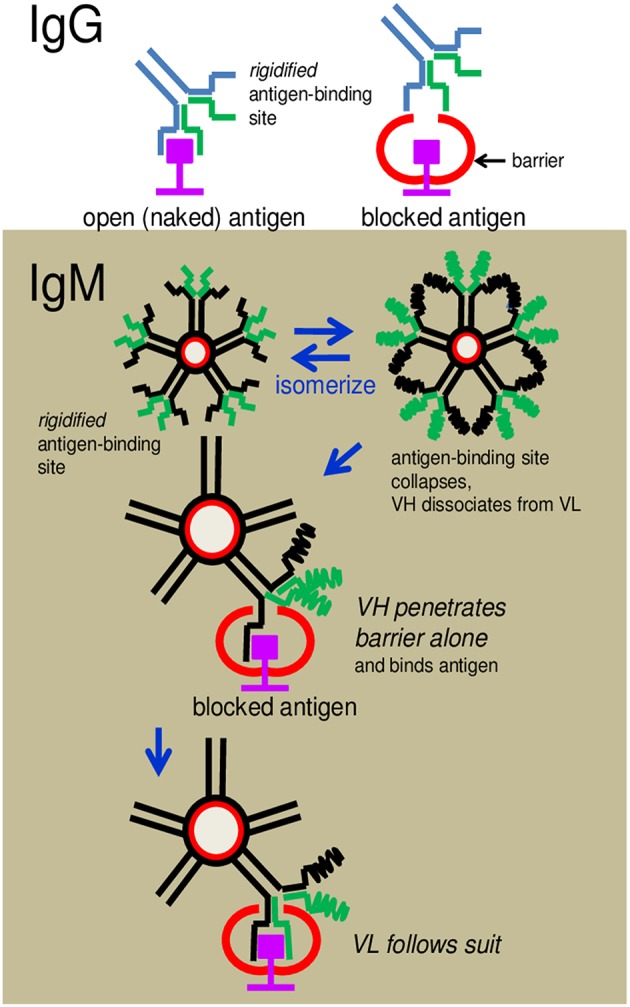
Cartoon illustrates how an IgG antibody is obstructed from binding to an antigen and how an IgM antibody overcomes the obstruction. **(Upper)** An IgG antibody is shown to dock, without problem, with an antigenic epitope displayed in an open environment, but is hindered when the antigen is blocked by a neighboring structure. The latter is due to the inflexibility (rigidity) of the pre-configured antigen-binding site. **(Lower)** IgM antibodies can circumvent the obstruction because the antigen-binding sites can dissociate to allow the separated VH or VL to wriggle through the obstacle independently and dock with the epitope.

The question thus arises: How do the IgM antibodies overcome obstacles and bind to cryptic antigens? The argument that the greater flexibility of the antigen-binding sites of these antibodies compared to those of IgG allows better penetrability is both vague and intangible. We propose, instead, a radically new mechanism of binding which is mechanistically and evolutionarily more tenable. We speculate that the antigen-binding sites of IgM exist in a state of constant flux between formation and collapse. This provides the opportunity for VH and VL to separate from each other frequently and probe for antigen independently. The advantage is, the individual polypeptide can wriggle through obstacles, if present, that otherwise obstruct the intact antigen-binding site (see Figure 9). If VH (or VL) is able to fold around the encountered antigen complementarily according to conformations permitted by the primary sequence, appropriate hydrophobic or electrostatic linkages can then form to secure anchorage. VL (or VH) follows suit immediately to complete the stranglehold on the antigen, the end-result being indistinguishable from the antigen-antibody complex formed by an IgG.

We base the hypothesis on several observations. First, there is precedence about single polypeptides acting as antibodies e.g., the ancestral “single-domain” proteins found in nurse shark and camelids, which are made entirely of heavy chains (46, 47). Many of these antibodies have long fingerlike CDR3s that enable the proteins to penetrate barriers and reach recessed antigens (48, 49). The HCDR3 projections of the “broadly neutralizing” IgG antibodies found in humans appear to operate in a similar fashion. Evolutionarily, as primordial proteins, present-day IgM antibodies may have retained this vestigial mode of antigen capture.

Second, there is evidence that polyreactive antibodies can isomerize frequently to adopt different conformations (14, 15) i.e., there is constant VH-VL dissociations and re-associations. We extend this activity to the immune IgMs in our hypothesis. Thus, there is a similarity between our hypothesis and the conformational flexibility theory for polyreactive antibodies (13, 15), the difference being whether a different conformation (isomer) is formed each time, or the same one always, and whether this formation happens before or during antigen contact. Most importantly, our hypothesis does not regard polyreactivity as an oddity but treats polyreactive and immune IgMs alike in a unifying hypothesis. In fact, our hypothesis predicts that IgM antibodies are more polyreactive than we know given the fact that, thanks to avidity, even weak interactions by VH or VL alone can result in stable binding.

It is not patently clear from our limited study whether, in the grand scheme, the proposed docking mechanism is a prerogative of all IgM antibodies regardless of their gross or fine specificity, or origin, or of IgM only. It is striking that both Mab23 and Mab50 are heavily mutated and yet both show weak affinities for the hapten. This can happen if there is a constant driving force separating VH from VL—with a strength no less than the affinity of Fv for the hapten. This can stem from CH1 or its association with CH2 (no hinge in-between) or CL (50, 51), particularly since CH1 can influence both the affinity and fine specificity of an antibody (52, 53), as well as the carrier-specificity (37). Exon-shuffling between an IgM and an IgG, or genetically engineering an IgG so it has no light chains, may help to substantiate our findings, but proving IgM docks using VH and VL separately is a more difficult proposition.

The findings lend support to the growing whispers that IgM antibodies or natural antibodies are beneficial to health (54–57). We can now appreciate this importance better knowing that these proteins not only appear to hunt effectively like an octopus with a myriad of highly flexible tentacles, but also recognize a much bigger universe of antigens than IgG—including antigens that are cryptic or even mutated. A tempting exploration of this knowledge is to supplement the later stage of an immune response with designer IgM antibodies when the infection persists unabatedly despite the presence of IgG antibodies.

## Data Availability

The raw data supporting the conclusions of this manuscript will be made available by the authors, without undue reservation, to any qualified researcher.

## Ethics Statement

1. All protocols dealing with animals were approved by the Animal Ethics Committee of the Chinese University of Hong Kong, Hong Kong.
2. Stored human sera kept in our routine laboratory were used according to The Joint Chinese University of Hong Kong – New Territories East Cluster Clinical Research Ethics Committee.

## Author Contributions

PL conceptualized the study, analyzed the data, and wrote the paper. DL, EL, KC, and NC produced the hybridomas and performed the initial antibody characterization. DL and FT performed the inhibition ELISA. EL, DL, and FT performed the IFA experiments and prepared the figures. EL prepared the hapten-protein conjugates. FT performed the antigen absorption studies and gene analysis. DL performed the WB analysis.

### Conflict of Interest Statement

FT and PL are currently employees at IgGENE, a tech-R&D company. EL, DL, FT, and PL filed a patent based on the preliminary findings of this study. The remaining authors declare that the research was conducted in the absence of any commercial or financial relationships that could be construed as a potential conflict of interest.

## References

[1] Schroeder HW Jr Cavacini L. Structure and function of immunoglobulins. J Allergy Clin Immunol. (2010) 125:S41–52. 10.1016/j.jaci.2009.09.046 20176268 PMC3670108

[2] Wedemayer GJ Patten PA Wang LH Schultz PG Stevens RC. Structural insights into the evolution of an antibody combining site. Science. (1997) 276:1665–9. 10.1126/science.276.5319.1665 9180069

[3] Schmidt AG Xu H Khan AR O'Donnell T Khurana S King LR. Preconfiguration of the antigen-binding site during affinity maturation of a broadly neutralizing influenza virus antibody. Proc Natl Acad Sci USA. (2013) 110:264–9. 10.1073/pnas.1218256109 23175789 PMC3538208

[4] Weiner GJ. Building better monoclonal antibody-based therapeutics. Nat Rev Cancer. (2015) 15:361–70. 10.1038/nrc3930 25998715 PMC4491443

[5] Landsteiner K. The Specificity of Serological Reactions. Springfield: Charles C Thomas (1936).

[6] Eigenbrot C Randal M Presta L Carter P Kossiakoff AA. X-ray structures of the antigen-binding domains from three variants of humanized anti-p185HER2 antibody 4D5 and comparison with molecular modeling. J Mol Biol. (1993) 229:969–95. 10.1006/jmbi.1993.1099 8095303

[7] Eisen HN Chakraborty AK. Evolving concepts of specificity in immune reactions. Proc Natl Acad Sci USA. (2010) 107:22373–80. 10.1073/pnas.1012051108 21173256 PMC3012479

[8] Dennison SM Stewart SM Stempel KC Liao HX Haynes BF Alam SM. Stable docking of neutralizing human immunodeficiency virus type 1 gp41 membrane-proximal external region monoclonal antibodies 2F5 and 4E10 is dependent on the membrane immersion depth of their epitope regions. J Virol. (2009) 83:10211–23. 10.1128/JVI.00571-09 19640992 PMC2748034

[9] Cauerhff A Braden BC Carvalho JG Aparicio R Polikarpov I Leoni J. Three-dimensional structure of the Fab from a human IgM cold agglutinin. J Immunol. (2000) 165:6422–8. 10.4049/jimmunol.165.11.6422 11086081

[10] Coutinho A Kazatchkine MD Avrameas S. Natural autoantibodies. Curr Opin Immunol. (1995) 7:812–8. 10.1016/0952-7915(95)80053-0 8679125

[11] Hardy RR B-1B cell development. J Immunol. (2006) 177:2749–54. 10.4049/jimmunol.177.5.2749 16920907

[12] New JS King RG Kearney JF. Manipulation of the glycan-specific natural antibody repertoire for immunotherapy. Immunol Rev. (2016) 270:32–50. 10.1111/imr.12397 26864103 PMC4755354

[13] Notkins AL. Polyreactivity of antibody molecules. Trends Immunol. (2004) 25:174–9. 10.1016/j.it.2004.02.004 15039043

[14] Foote J Milstein C. Conformational isomerism and the diversity of antibodies. Proc Natl Acad Sci USA. (1994) 91:10370–4. 10.1073/pnas.91.22.10370 7937957 PMC45021

[15] James LC Roversi P Tawfik DS. Antibody multispecificity mediated by conformational diversity. Science. (2003) 299:1362–7. 10.1126/science.1079731 12610298

[16] Yan M Tam FC Kan B Lim PL. Combined rapid (TUBEX) test for typhoid-paratyphoid A fever based on strong anti-O12 response: design and critical assessment of sensitivity. PLoS ONE. (2011) 6:e24743. 10.1371/journal.pone.0024743 21935450 PMC3174194

[17] Niu H Leung DT Ma CH Law EC Tam FC Lim PL. Cells that produce deleterious autoreactive antibodies are vulnerable to suicide. J Immunol. (2008) 181:2246–57. 10.4049/jimmunol.181.3.2246 18641365

[18] Ma CH Hui J Tang JT Leung DT Chui YL Fok TF. Antibodies to guanosine triphosphate misidentified as anti-double-stranded DNA antibodies in a patient with antinuclear antibody-negative lupus, due to buckling of insolubilized assay DNA. Arthritis Rheum. (2004) 50:1533–8. 10.1002/art.20188 15146423

[19] Cherfils J Zeghouf M. Regulation of small GTPases by GEFs, GAPs, and GDIs. Physiol Rev. (2013) 93:269–309. 10.1152/physrev.00003.2012 23303910

[20] Erlanger BF Beiser SM. Antibodies specific for ribonucleosides and ribonucleotides and their reaction with DNA. Proc Natl Acad Sci USA. (1964) 52:68–74. 10.1073/pnas.52.1.68 14192660 PMC300575

[21] Kavran JM Leahy DJ. Coupling antibody to cyanogen bromide-activated sepharose. Methods Enzymol. (2014) 541:27–34. 10.1016/B978-0-12-420119-4.00003-3 24674060 PMC4337859

[22] Colburn KK Wong AL Weisbart RH Green LM. Antiguanosine antibodies in murine and human lupus have the internal image of G-binding proteins. J Rheumatol. (2003) 30:993–7. 12734894

[23] Orosz F Ovadi J. A simple method for the determination of dissociation constants by displacement ELISA. J Immunol Methods. (2002) 270:155–62. 10.1016/S0022-1759(02)00295-8 12379321

[24] Nogales E Wolf SG Downing KH. Structure of the alpha beta tubulin dimer by electron crystallography. Nature. (1998) 391:199–203. 10.1038/34465 9428769

[25] Czajkowsky DM Shao Z. The human IgM pentamer is a mushroom-shaped molecule with a flexural bias. Proc Natl Acad Sci USA. (2009) 106:14960–5. 10.1073/pnas.0903805106 19706439 PMC2736442

[26] Klein JS Bjorkman PJ. Few and far between: how HIV may be evading antibody avidity. PLoS Pathog. (2010) 6:e1000908. 10.1371/journal.ppat.1000908 20523901 PMC2877745

[27] Hattori T Lai D Dementieva IS Montano SP Kurosawa K Zheng Y. Antigen clasping by two antigen-binding sites of an exceptionally specific antibody for histone methylation. Proc Natl Acad Sci USA. (2016) 113:2092–7. 10.1073/pnas.1522691113 26862167 PMC4776465

[28] Pertschuk LP. Immunofluorescence of soft-tissue tumors with anti-smooth-muscle and anti-skeletal-muscle antibodies. Am J Clin Pathol. (1975) 63:332–42. 10.1093/ajcp/63.3.332 1090146

[29] Zhu J Bilan PJ Moyers JS Antonetti DA Kahn CR. Rad, a novel Ras-related GTPase, interacts with skeletal muscle beta-tropomyosin. J Biol Chem. (1996) 271:768–73. 10.1074/jbc.271.2.768 8557685

[30] Chesebro B Bloth B Svehag SE. The ultrastructure of normal and pathological IgM immunoglobulins. J Exp Med. (1968) 127:399–410. 10.1084/jem.127.3.399 4169962 PMC2138459

[31] Uehata M Ishizaki T Satoh H Ono T Kawahara T Morishita T. Calcium sensitization of smooth muscle mediated by a Rho-associated protein kinase in hypertension. Nature. (1997) 389:990–4. 10.1038/40187 9353125

[32] Sanden C Broselid S Cornmark L Andersson K Daszkiewicz-Nilsson J Martensson UE. G protein-coupled estrogen receptor 1/G protein-coupled receptor 30 localizes in the plasma membrane and traffics intracellularly on cytokeratin intermediate filaments. Mol Pharmacol. (2011) 79:400–10. 10.1124/mol.110.069500 21149639

[33] Hall A. G proteins and small GTPases: distant relatives keep in touch. Science. (1998) 280:2074–5. 10.1126/science.280.5372.2074 9669963

[34] Sasson Y Navon-Perry L Huppert D Hirsch JA. RGK family G-domain:GTP analog complex structures and nucleotide-binding properties. J Mol Biol. (2011) 413:372–89. 10.1016/j.jmb.2011.08.017 21903096

[35] Qiao S Luo QS Zhao Y Zhang XJC Huang YH. Structural basis for lipopolysaccharide insertion in the bacterial outer membrane. Nature. (2014) 511:108–U523. 10.1038/nature13484 24990751

[36] Lim PL Leung DT Chui YL Ma CH. Structural analysis of a phosphorylcholine-binding antibody which exhibits a unique carrier specificity for Trichinella spiralis. Mol Immunol. (1994) 31:1109–16. 10.1016/0161-5890(94)90106-6 7935500

[37] Tam FC Ma CH Leung DT Sutton B Lim PL. Carrier-specificity of a phosphorylcholine-binding antibody requires the presence of the constant domains and is not dependent on the unique VH49 glycine or VH30 threonine residues. J Immunol Methods. (2007) 321:152–63. 10.1016/j.jim.2007.01.013 17331532

[38] Lee JH Andrabi R Su CY Yasmeen A Julien JP Kong L. A broadly neutralizing antibody targets the dynamic HIV envelope trimer apex via a long, rigidified, and anionic beta-hairpin structure. Immunity. (2017) 46:690–702. 10.1016/j.immuni.2017.03.017 28423342 PMC5400778

[39] Sompuram SR Vani K Messana E Bogen SA. A molecular mechanism of formalin fixation and antigen retrieval. Am J Clin Pathol. (2004) 121:190–9. 10.1309/BRN7CTX1E84NWWPL 14983931

[40] Leung DT Ma CH Niu H Liew CT Tang JT Lim PL. Nuclear telomerase is less accessible to antibody probing than known nuclear antigens: retrieval with new immunostaining buffer. Histochem Cell Biol. (2005) 123:105–12. 10.1007/s00418-004-0721-x 15538612

[41] Denda T Kamoshida S Kawamura J Harada K Kawai K Kuwao S. Optimal antigen retrieval for ethanol-fixed cytologic smears. Cancer Cytopathol. (2012) 120:167–76. 10.1002/cncy.21192 22434540

[42] van Gils MJ Bunnik EM Boeser-Nunnink BD Burger JA Terlouw-Klein M Verwer N. Longer V1V2 region with increased number of potential N-linked glycosylation sites in the HIV-1 envelope glycoprotein protects against HIV-specific neutralizing antibodies. J Virol. (2011) 85:6986–95. 10.1128/JVI.00268-11 21593147 PMC3126602

[43] Pejchal R Doores KJ Walker LM Khayat R Huang PS Wang SK. A potent and broad neutralizing antibody recognizes and penetrates the HIV glycan shield. Science. (2011) 334:1097–103. 10.1126/science.1213256 21998254 PMC3280215

[44] Klasse PJ Sattentau QJ. Occupancy and mechanism in antibody-mediated neutralization of animal viruses. J Gen Virol. (2002) 83:2091–108. 10.1099/0022-1317-83-9-2091 12185262

[45] Tam FC Lim PL The TUBEX typhoid test based on particle-inhibition immunoassay detects IgM but not IgG anti-O9 antibodies. J Immunol Methods. (2003) 282:83–91. 10.1016/j.jim.2003.07.006 14604543

[46] Marchalonis JJ Schluter SF Bernstein RM Hohman VS. Antibodies of sharks: revolution and evolution. Immunol Rev. (1998) 166:103–122. 10.1111/j.1600-065X.1998.tb01256.x 9914906

[47] Conrath KE Wernery U Muyldermans S Nguyen VK. Emergence and evolution of functional heavy-chain antibodies in Camelidae. Dev Comp Immunol. (2003) 27:87–103. 10.1016/S0145-305X(02)00071-X 12543123

[48] Stijlemans B Conrath K Cortez-Retamozo V Van Xong H Wyns L Senter P. Efficient targeting of conserved cryptic epitopes of infectious agents by single domain antibodies. African trypanosomes as paradigm. J Biol Chem. (2004) 279:1256–61. 10.1074/jbc.M307341200 14527957

[49] Acharya P Luongo TS Georgiev IS Matz J Schmidt SD Louder MK. Heavy chain-only IgG2b llama antibody effects near-pan HIV-1 neutralization by recognizing a CD4-induced epitope that includes elements of coreceptor- and CD4-binding sites. J Virol. (2013) 87:10173–81. 10.1128/JVI.01332-13 23843638 PMC3753989

[50] Rothlisberger D Honegger A Pluckthun A. Domain interactions in the Fab fragment: a comparative evaluation of the single-chain Fv and Fab format engineered with variable domains of different stability. J Mol Biol. (2005) 347:773–89. 10.1016/j.jmb.2005.01.053 15769469

[51] Wang N Smith WE Miller BR Aivazian D Lugovskoy AA Reff ME. Conserved amino acid networks involved in antibody variable domain interactions. Prot Struct Funct Bioinform. (2009) 76:99–114. 10.1002/prot.22319 19089973

[52] Pritsch O HudryClergeon G Buckle M Petillot Y Bouvet JP Gagnon J. Can immunoglobulin C(H)1 constant region domain modulate antigen binding affinity of antibodies? J Clin Invest. (1996) 98:2235–43. 10.1172/JCI119033 8941639 PMC507672

[53] Torres M Fernandez-Fuentes N Fiser A Casadevall A. The immunoglobulin heavy chain constant region affects kinetic and thermodynamic parameters of antibody variable region interactions with antigen. J Biol Chem. (2007) 282:13917–27. 10.1074/jbc.M700661200 17353196

[54] Boes M Prodeus AP Schmidt T Carroll MC Chen JZ. A critical role of natural immunoglobulin M in immediate defense against systemic bacterial infection. J Exp Med. (1998) 188:2381–6. 10.1084/jem.188.12.2381 9858525 PMC2212438

[55] Ehrenstein MR Notley CA. The importance of natural IgM: scavenger, protector and regulator. Nat Rev Immunol. (2010) 10:778–86. 10.1038/nri2849 20948548

[56] Baumgarth N. How specific is too specific? B-cell responses to viral infections reveal the importance of breadth over depth. Immunol Rev. (2013) 255:82–94. 10.1111/imr.12094 23947349 PMC3748619

[57] Wang H Coligan JE Morse HC III. Emerging functions of natural IgM and its Fc receptor FCMR in immune homeostasis. Front Immunol. (2016) 7:99. 10.3389/fimmu.2016.00099 27014278 PMC4791374

